# A semi-automated pipeline integrating ImageJ/Fiji and StarDist for the reproducible quantification of cellular and optical density metrics in immunofluorescence images of brain tissue

**DOI:** 10.3389/fnana.2026.1778296

**Published:** 2026-03-27

**Authors:** Sandra Isabel Marques, Helena Carmo, Félix Carvalho, João Pedro Silva, Susana Isabel Sá

**Affiliations:** 1 UCIBIO—Applied Molecular Biosciences Unit, Laboratory of Toxicology, Faculty of Pharmacy, University of Porto, Porto, Portugal; 2 i4HB—Institute for Health and Bioeconomy, Faculty of Pharmacy, University of Porto, Porto, Portugal; 3 RISE-HEALTH, Faculty of Medicine, University of Porto, Porto, Portugal

**Keywords:** automated image analysis, cell quantification, ImageJ (FIJI), immunofluorescence quantification, spreadsheet software, StarDist

## Abstract

Quantitative immunofluorescence is widely used to assess molecular expression and cellular distribution across biological tissues, yet the analysis of large image datasets remains time-consuming and prone to user-dependent variability. To address these limitations, we herein developed a semi-automated workflow that integrates ImageJ/Fiji for image processing, StarDist for nuclear segmentation, and spreadsheet- or Python-based routines for data curation. The pipeline standardizes critical analytical steps, including scale calibration, region-of-interest (ROI) definition, channel selection, and z-stack handling, while preserving essential metadata through a structured file-naming system. Optical density and cell-number metrics are exported automatically in a consistent format, enabling efficient consolidation into a unified dataset. Subsequent curation can be performed either manually in a spreadsheet software or fully automatically through custom Python scripts, allowing extraction of sample identifiers, regions, and markers, as well as calculation of normalized intensity values. Comparison with existing protocols proved that this workflow adheres to widely accepted quantification principles while markedly improving reproducibility, consistency, and analytical throughput. This method offers a straightforward, transparent, and scalable solution for fluorescence-based quantification suitable for laboratories with varying levels of computational expertise.

## 1 Introduction

Immunofluorescence (IF) is a widely applied technique for the detection and visualization of specific molecules within biological samples (Ramos-Vara and Miller, 2014). By combining antibodies coupled with fluorophores, IF leverages the high specificity of antibody-antigen interactions, enabling both qualitative and quantitative assessments of protein expression and localization across tissues and cell populations (Ramos-Vara and Miller, 2014). Its versatility has made it a cornerstone in neurobiology, pharmacology, and pathology, where spatial information and quantitative accuracy are equally important.

Robust statistical analysis requires adequate sample size and coverage, which inevitably generates large datasets. For instance, in experiments involving rodents, evaluating a single brain region may require at least five to six animals per treatment group, with multiple sections and fields of view acquired per animal. Such an approach easily results in hundreds to thousands of images (Barker and Ullian, 2010). Notably, a typical experimental design may involve analyzing more than a single target across several regions, and such analysis is usually complemented by nuclear staining with 4’,6-diamidino-2-phenylindole (DAPI) to provide reference values for cell density and number. This design, although biologically comprehensive, produces extensive image sets that pose a major challenge for consistent and efficient quantification.

To address this challenge, we developed a streamlined workflow that integrates widely available tools, such as ImageJ/Fiji for image processing, StarDist for automated nuclear segmentation, and a spreadsheet software-based platform for data handling. Following image acquisition, we standardized the images’ scale, defined ROIs, and quantified signal intensity (optical density, OD) across different channels. Nuclear quantification was performed using StarDist, a machine-learning-based tool that provides robust and reproducible cell counts (Schmidt et al., 2018). Automated prompts ensured that file naming was retained during file export, thereby linking image identifiers with their corresponding entries in the dataset. This organization facilitated the use of Pivot tables and filters within the spreadsheet software, enabling rapid generation of summary statistics and visualization outputs, such as pivot-based heatmaps and comparative charts.

While this approach does not primarily aim at introducing new biological findings, it clearly provides a reproducible and practical framework for IF imaging post-processing, specifically through different endpoints’ quantification. By combining established tools into a coherent pipeline, we aim to minimize variability, accelerate data processing and handling, and improve reproducibility, ultimately making IF-based studies more accessible and transparent. This macro iteratively processes each image stack, identifies the focal range, applies user-defined ROIs, executes StarDist segmentation, and exports OD measurements for all channels. Importantly, the present work does not aim to introduce a novel biological assay or to provide absolute quantification of protein abundance. Instead, it describes a practical and reproducible image analysis workflow for relative quantification of cellular and OD metrics in immunofluorescence images, based on widely available and open-source tools. By focusing on standardization, scalability, and ease of implementation, this pipeline is intended to be readily adopted by researchers with varying levels of computational expertise, facilitating consistent analysis of large IF datasets across laboratories.

Importantly, the present work does not introduce a novel segmentation algorithm or redefine fluorescence quantification principles. Rather, it integrates established tools, such as ImageJ/Fiji, for image processing (Schindelin et al., 2012; Schroeder et al., 2021), StarDist for deep learning–based nuclear segmentation (Schmidt et al., 2018) and structured spreadsheets or Python-based routines for data consolidation, into a standardized workflow designed to enhance consistency and scalability across large immunofluorescence datasets. Several recently published Fiji/ImageJ-based tools focus on automated cell counting or morphometric profiling, including macro-based automated counting approaches such as AutoCount (Sharara et al., 2025), machine-learning–based segmentation workflows such as ACCT (Kataras et al., 2023), and broader morphometric analysis frameworks such as Lusca (Šimunić et al., 2024). While these tools provide validated segmentation and quantification strategies, they differ in scope from the present workflow, which harmonizes z-stack handling, ROI definition (including dual-region logic), multi-channel optical density extraction, and metadata-preserving export within a unified processing structure. A structured comparison of representative fluorescence quantification tools and the present workflow is provided in Supplementary Table 1. The objective is therefore the operational standardization and reproducible dataset generation, rather than algorithmic innovation.

## 2 Materials and methods

### 2.1 Samples

This work utilized previously collected brain sections from twenty young-adult female Wistar rats (*Rattus norvegicus*) from an independent study conducted in the accredited animal facility at the Faculty of Medicine of the University of Porto (FMUP). All procedures were performed in accordance with Portuguese legislation on animal welfare (Decree-Law 113/2013) and the European Directive 2010/63/EU. All procedures had been reviewed and approved by FMUP’s institutional animal welfare and ethics committee (ORBEA). For the present study, only the archived histological sections derived from that approved work were used; no additional animal procedures were performed.

Following perfusion with saline and fixation in 4% paraformaldehyde, the brains were removed, rinsed three times in phosphate buffer (10 min each), and post-fixed in the same fixative for 30 min. Tissues were then cryoprotected by sequential immersion in sucrose solutions with increasing concentrations from 10 up to 30%, before storage at −20°C in OLMOS solution [0.95% *(w/v)* Na_2_HPO_4_⋅2H_2_O, 0.212% *(w/v)* NaH_2_PO_4_⋅H_2_O, 30% *(w/v)* sucrose, 1% *(w/v)* polyvinylpyrrolidone (PVP), 30% *(v/v)* ethylene glycol]. For IF, brains were bisected along the mid-sagittal plane, and coronal sections (35 μm) were obtained using a vibratome, as previously described (Dias-Carvalho et al., 2022; Sá and Madeira, 2012).

### 2.2 Immunofluorescence

IF was performed as previously described (Neves et al., 2025; Sá and Madeira, 2012). Briefly, sections were recovered from OLMOS solution, rinsed in 0.01 M phosphate-buffered saline (PBS), and incubated in blocking solution containing 5% normal horse serum (Vector Laboratories). Free-floating sections were then incubated with primary antibodies at 4°C for 72 h in PBS supplemented with 0.5% Triton X-100, using dilutions optimized for each antibody. Following incubation, sections were mounted on gelatin-coated slides and processed at room temperature. Secondary antibody incubation was carried out for 1 h using Alexa Fluor™ 488, (S11223, Invitrogen, Carlsbad, CA, United States), and horse anti-mouse Dylight™ 594, (DI-2594, Vector Laboratories, CA, United States) (1:1,000), protected from light, in a humidified chamber. Nuclear counterstaining was performed with DAPI (ThermoFisher Scientific) (1:100), and sections were mounted with Histomount medium (National Diagnostics, Atlanta, GA, United States).

### 2.3 Image acquisition

Photomicrographs were obtained from selected brain regions, including the hippocampal formation, prefrontal cortex, and nucleus accumbens, using a Carl Zeiss Axio Imager 2.0 microscope (Zeiss, NY, United States) equipped with a digital color camera and controlled by AxioVision Rel. 4.8 software. To ensure reproducibility, images for each target protein were captured under identical acquisition parameters, including exposure time, gain, offset, and z-stack spacing. Fluorescent signals were detected using filter sets with excitation/emission wavelengths of 365/445 nm (DAPI), 470/525 nm (DyLight and Alexa Fluor 488), and 565/620 nm (Texas Red and Alexa Fluor 594). Images were acquired with a 20 × objective in manual scanning mode and exported in TIFF format for subsequent analysis.

### 2.4 Workflow overview

The following section provides a step-by-step description of the workflow setup and execution. It covers the installation of required add-ons in ImageJ, the organization of the data folder structure, and the use of the custom macro to perform automated image processing and data export.

#### 2.4.1 Code availability

Photomicrograph analysis was performed using ImageJ (FIJI) software^1^ in combination with the ElmFlouLzCount.ijm macro.^2^ Subsequent datasheet processing was conducted using Excel or equivalent spreadsheet software. A Python script implemented in Google Colab, provides an automated alternative for data consolidation and analysis, and is also available in the associated GitHub repository.^3^

#### 2.4.2 Folder structure and macro initiation

To enable seamless downstream analysis, a standardized folder hierarchy was implemented to maintain consistent organization and facilitate automated labeling during data export. The structure consists of four nested levels:

- Level 1: root directory containing all samples.
- Level 2: individual folders for each sample.
- Level 3: subfolders corresponding to the different regions analyzed.
- Level 4: subfolders containing the image stacks generated by the microscope software.

A confirmation step, requiring the user’s acceptance, was added upon launching the macro to ensure this structure is established. After acceptance, the macro progresses, prompting the user to specify the root directory containing the image files. During initialization, the user selects the Level 1 directory. This design ensures that processed outputs from each image retain a unique identifier derived from the nested folder names. Each analyzed image produces two data files, which are automatically labeled in the format [Level2]_[Level3]_[Level4]_results. For example, if the root directory (Level 1) contains an animal folder named *111* (Level 2), with a brain region subfolder *REGION* (Level 3), and an acquisition folder *PROT1-PROT2-2025 0033* (Level 4), the exported files will be labeled as: 111_REGION_PROT1-PROT2-2025-0033_results.

This structured naming convention preserves the experimental context (animal → brain region → acquisition set) and streamlines downstream data handling and filtering in a spreadsheet software. The results are saved in the same location where the Level 1 folder is stored as “Results_Level1.”

#### 2.4.3 Parameter selection

**FIGURE 1:**
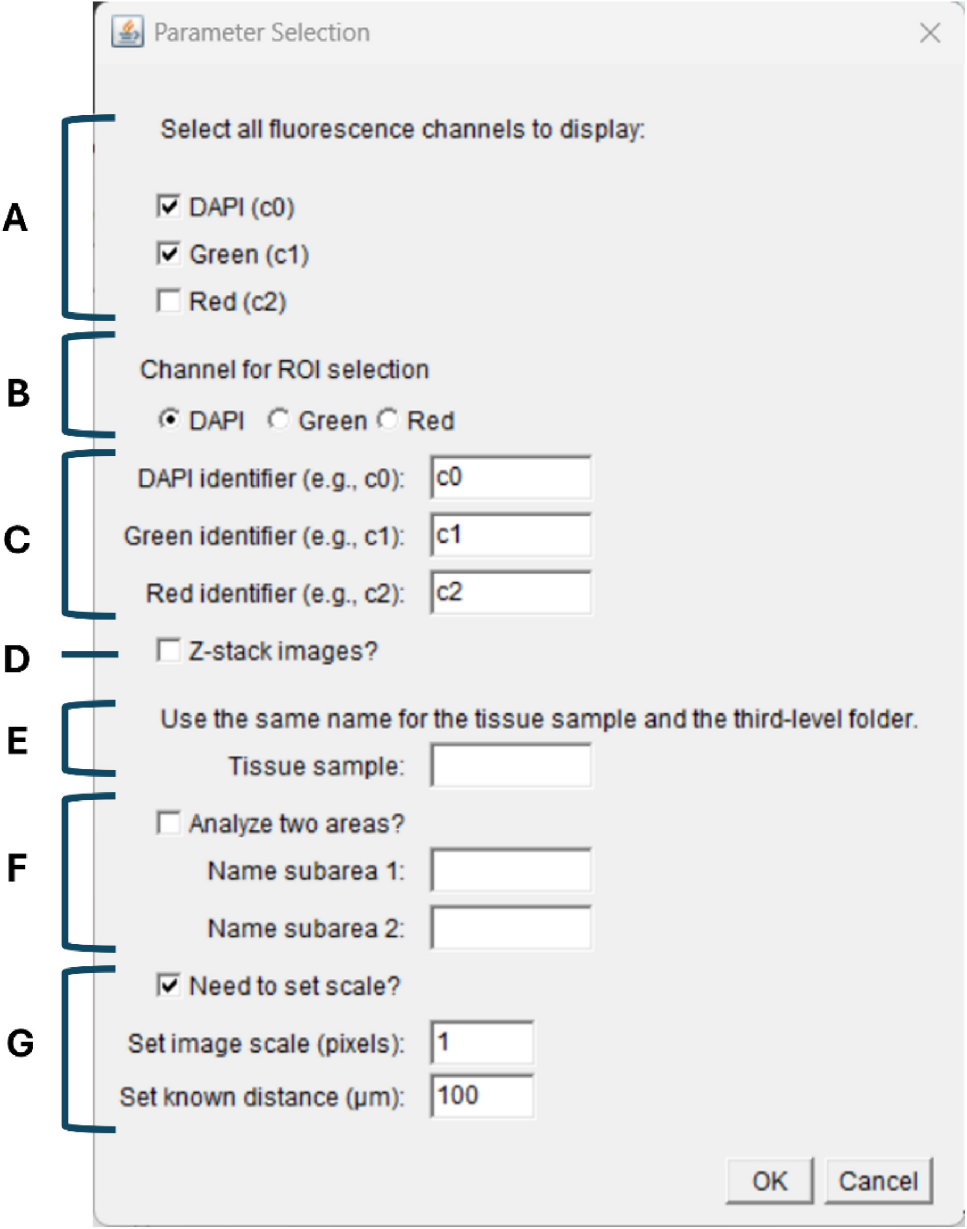
Parameter Selection interface used to define image processing settings. The interface prompts the user to specify all analytical parameters before initiating image processing. The interface includes options to **(A)** select the fluorescence channels to be analyzed (DAPI, green, and red), **(B)** designate the channel displayed during ROI definition, and **(C)** assign channel identifiers compatible with the microscope software. Additional settings allow the user to **(D)** indicate whether the input files are z-stacks, **(E)** assign a standardized tissue sample name corresponding to the Level-3 folder structure, and **(F)** enable dual-region analysis when an image contains two anatomical areas. A final section **(G)** enables scale calibration by defining pixel size and known distance, ensuring uniform measurement parameters across all images processed within the session.

After directory selection, the user is presented with a parameter-selection interface (Figure 1). Each option within this prompt defines a specific aspect of the analysis workflow:

- A: Channel selection
  Defines the fluorescence channels to be included in the analysis. Multiple channels (c0, c1, c2) may be selected simultaneously. Channel 0 (c0) corresponds to the DAPI signal (blue; 365/445 nm), Channel 1 (c1) to the green fluorophore (DyLight 488 / Alexa Fluor 488; 470/525 nm), and Channel 2 (c2) to the red fluorophore (Texas Red / Alexa Fluor 594; 565/620 nm).
- B: ROI display channel
  Specifies the channel used during image visualization for ROI definition. This channel is displayed during the streaming step, where the user confirms whether the full image should be analyzed or if trimming is required.
- C: Channel naming standardization
  Harmonizes naming conventions derived from the microscope software to ensure consistent identification of each channel during export and analysis.
- D: Image type (z-stack or single plane)
  Indicates whether the input files are z-stacks or single images, allowing the macro to apply the appropriate processing sequence.
- E: Region selection
  Defines the brain region or the tissue sample (if not brain) to be analyzed. The macro processes images from one region at a time based on this selection, corresponding to Level 3 of folder organization. Based on this selection, the macro retrieves all images corresponding to the chosen region from the input directory, across all samples, and processes them sequentially. This automated retrieval ensures that every relevant image is included in the analysis without the need for manual file selection.
- F: Dual-region analysis (optional)
  Used only when a single image contains two anatomical regions. Activating this option enables simultaneous quantification of both regions following independent ROI definition.
- G: Scale definition
  Sets the image scale, which is uniformly applied to all subsequent images to ensure consistent measurement parameters across the dataset.

#### 2.4.4 Image processing

**FIGURE 2:**
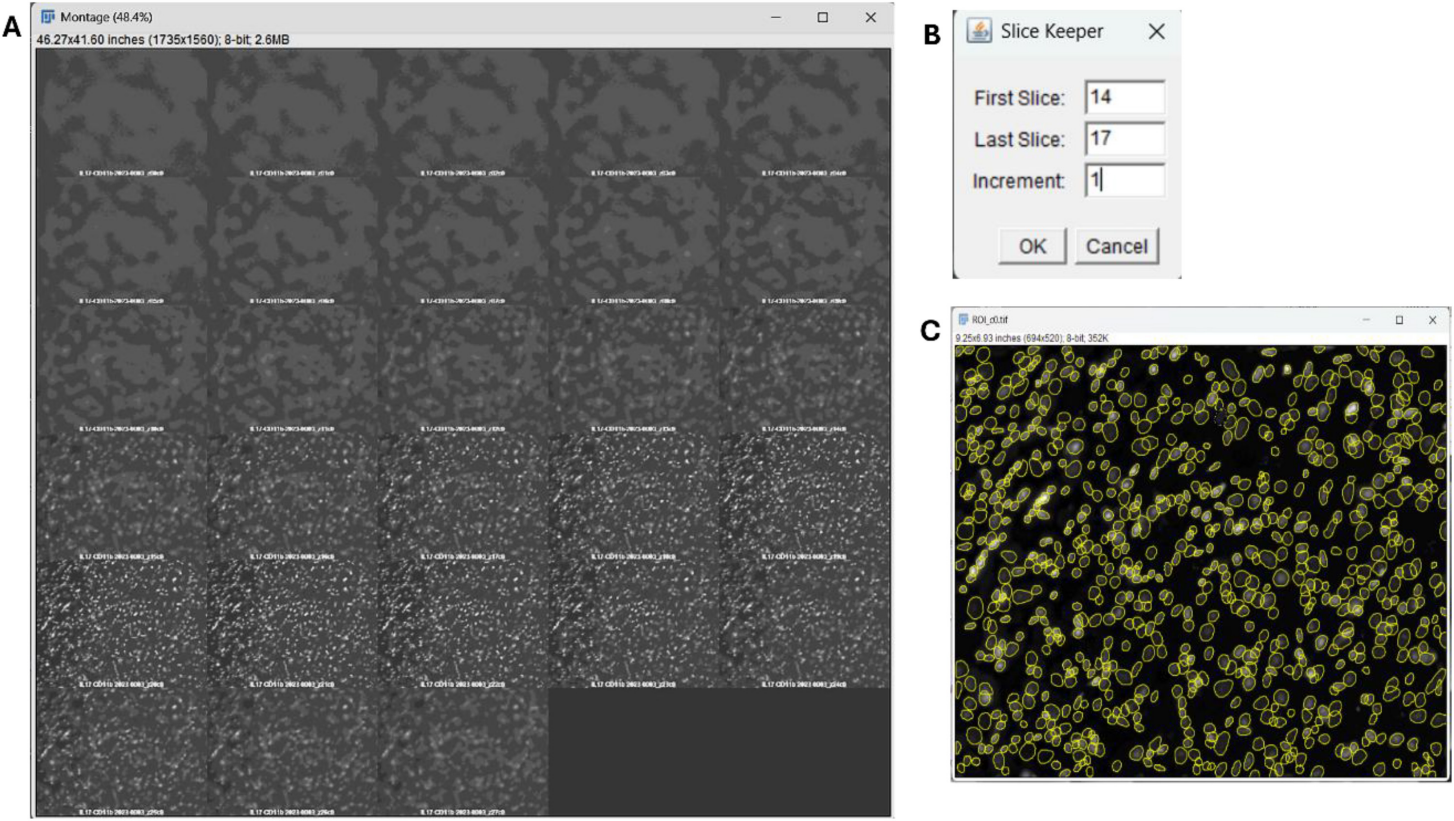
Selection of in-focus slices and automated nucleus detection. The macro first displays each z-stack as a tiled montage **(A)**, allowing visual inspection of all planes and identification of the in-focus range. The Slice Keeper interface **(B)** is then used to specify the first and last slices to retain, with the increment set to 1 to ensure continuous coverage. After ROI definition, StarDist is applied to the DAPI channel (c0) to detect and segment all nuclei within the selected region **(C)**, producing the mask used for quantification.

After parameter settings, and selection of images in z-stack, the macro guides the user through the following sequence of steps:

- Stack visualization
  Each image stack is presented in a tiled display (Figure 2A), allowing the user to inspect all planes simultaneously. The *Brightness & Contrast* tool in ImageJ may be used to optimize visualization and assist in identifying the in-focus slices. However, if used, one cannot press the “Apply” button. This step is essential, as the subsequent prompt opens a slice-selection interface in which the user specifies the first and last images within the focal range (Figure 2B). The increment should be set to 1, ensuring continuous coverage of all relevant focal planes.
- Composite image generation and ROI selection
  The macro creates a composite image from the selected stack. The stacked image is presented using the channel previously decided in the parameters, and the user is prompted to define the region of interest (ROI) if the full field of view is not required. Standard selection tools (rectangle or polygon) can be used.
- Cell detection with StarDist
  StarDist is automatically executed on the DAPI identifier channel to detect and quantify all nuclei within the defined ROI (Figure 2C). The resulting measurements are saved as a.*csv* file in the previously defined input/output directory, using the standardized naming format:
[L⁢e⁢v⁢e⁢l⁢2]⁢_⁢[L⁢e⁢v⁢e⁢l⁢3]⁢_⁢[L⁢e⁢v⁢e⁢l⁢4]⁢_⁢r⁢e⁢s⁢u⁢l⁢t⁢s⁢_⁢D⁢A⁢P⁢I.
  Although StarDist is a costumizerd tool, the specific settings programmed, present on the macro, are:
  Run [“Command From Macro,” “command = (de.csbdresden.stardist.StarDist2D), args = (“input”:“ROI_Sub Area1.tif,” “modelChoice”:“Versatile (fluorescent nuclei),” “normalizeInput”:“true,” “percentileBottom”:“1.0,” “percentile Top”:“99.8,” “probThresh”:“0.5,” “nmsThresh”:“0.4,” “output Type”:“Both,” “nTiles”:“1,” “excludeBoundary”:“2,” “roiPosition”: “Automatic,” “verbose”:“false,” “showCsbdeepProgress”: “false,” “showProbAndDist”:(“false”), process = (“false)”];
  To verify the reliability of the automated nuclear detection, StarDist-based quantification was validated against manual nuclear counts performed on a representative subset of images, as detailed in Supplementary Information (Manual Quantification).
- ROI confirmation and quantification
  The composite image is displayed for ROI verification. This should be left unmodified if no changes are needed. A prompt confirms the ROI selection.
  The macro then measures the OD across all selected channels (c0, c1, c2). Results are stored as.*csv* files, properly identified in the output directory as [Level2]_[Level3]_[Level4]_results_DO, and the macro automatically advances to the next image.
  If the image is a single plane with no z-stack, the macro bypasses the focal-plane selection step and proceeds directly to ROI definition. The analysis then continues following the same sequence of operations described above.
- Special case: multiple brain regions in a single image
  If a single image contains two anatomical regions (e.g., dentate gyrus and hilus), both regions are specified in the initial interface prompt (Figure 1F). The macro then follows the standard workflow (Steps 1–3), but Step 4 includes an additional ROI definition stage. The first selection defines the overall ROI, and the second delineates Area 1 using the polygon or any cutting tool. Area 2 is automatically defined as the remaining portion of the ROI. Quantification is then performed independently for each region. The names of Area 1 and Area 2 are incorporated into Level 3 of the output file naming structure.

## 3 Results and data curation workflow

The macro generates two output files per image: one containing the OD and area measurements for the selected channels, and another containing the StarDist-derived nuclear counts. These files follow the naming structure described previously, with OD quantification saved as *[Level2]_[Level3]_[Level4]_results_DO* and StarDist quantification saved as *[Level2]_[Level3]_[Level4]_results_DAPI*.

Two equivalent approaches can be used for data analysis, both leading to the same final dataset. The first is a user-guided workflow performed in a spreadsheet software, relying on built-in functions for data extraction and organization. The second approach employs custom Python scripts that replicate these operations automatically, consolidating and processing all output files with minimal user intervention.

### 3.1 Optical density user data curation

ImageJ’s macro generates two data sheets for each processed image, which can become impractical when working with large datasets. To streamline the OD analysis, the first step involves running a simple Python script that consolidates all individual. *csv* files into a single table. Because the labeling system is highly structured, merging the entries does not compromise data integrity; the sample, target, and brain region are fully encoded in the file name and therefore do not require manual annotation.

**FIGURE 3:**
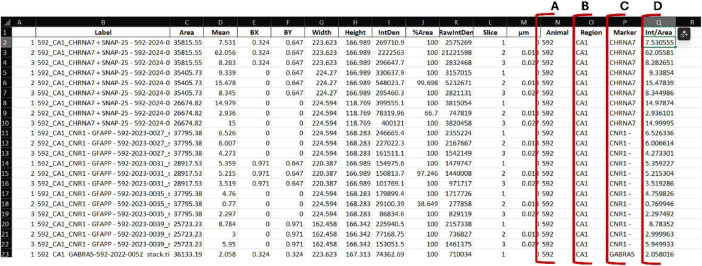
Consolidated worksheet after metadata extraction and intensity per area calculation. Each row represents a single ROI-level quantification extracted from the ImageJ macro outputs. Columns include the original image label, geometric measurements (Area, Width, Height), OD values (IntDen, RawIntDen), and additional metadata derived from the file naming structure. The automatically extracted identifiers: **(A)** Animal (Level 2), **(B)** Region (Level 3), **(C)** and Marker (Level 4), are shown outlined in red. A calculated column **(D)** Int/Area is added to derive intensity per unit area by dividing the IntDen value by the corresponding Area value, enabling normalized comparisons across images.

The resulting consolidated table may contain more than 5,000 entries, but with appropriate filtering, downstream analysis remains straightforward (Figure 3). We recommend duplicating the consolidated worksheet to retain an unmodified raw version as a backup. The next step is to extract the relevant metadata from the file label, specifically the Level 2, Level 3, and, when applicable, Level 4 identifiers. In the example shown in Figure 3, these correspond to the animal number (Level 2, Figure 3A), brain region (Level 3, Figure 3B), and marker (Level 4, when included in the original file name, Figure 3C). This can be achieved using the spreadsheet functions “LEFT,” “MID,” or, more efficiently, “TEXTSPLIT” with “_” defined as the column delimiter. When StarDist is used solely for quantifying the number of cells, and the intensity of the DAPI channel is not required for downstream analysis, the corresponding entries can be removed by filtering the dataset by channel (e.g., c0) and deleting those rows. Upon data filtering, an additional column is required to calculate the parameter of interest, *intensity per area*, obtained by dividing the values in the *IntDen* column by those in the *Area* column (Figure 3D).

The final organization of the dataset, prior to exporting to the statistical analysis software, is achieved through a pivot table. This can be generated by selecting all entries in the consolidated worksheet and using the *Insert → PivotTable* command, preferably placing the output in a new worksheet for clarity. The *PivotTable* field menu enables users to define the table structure by dragging available fields, each corresponding to a column in the original dataset, into the rows, columns, and value areas.

**FIGURE 4:**
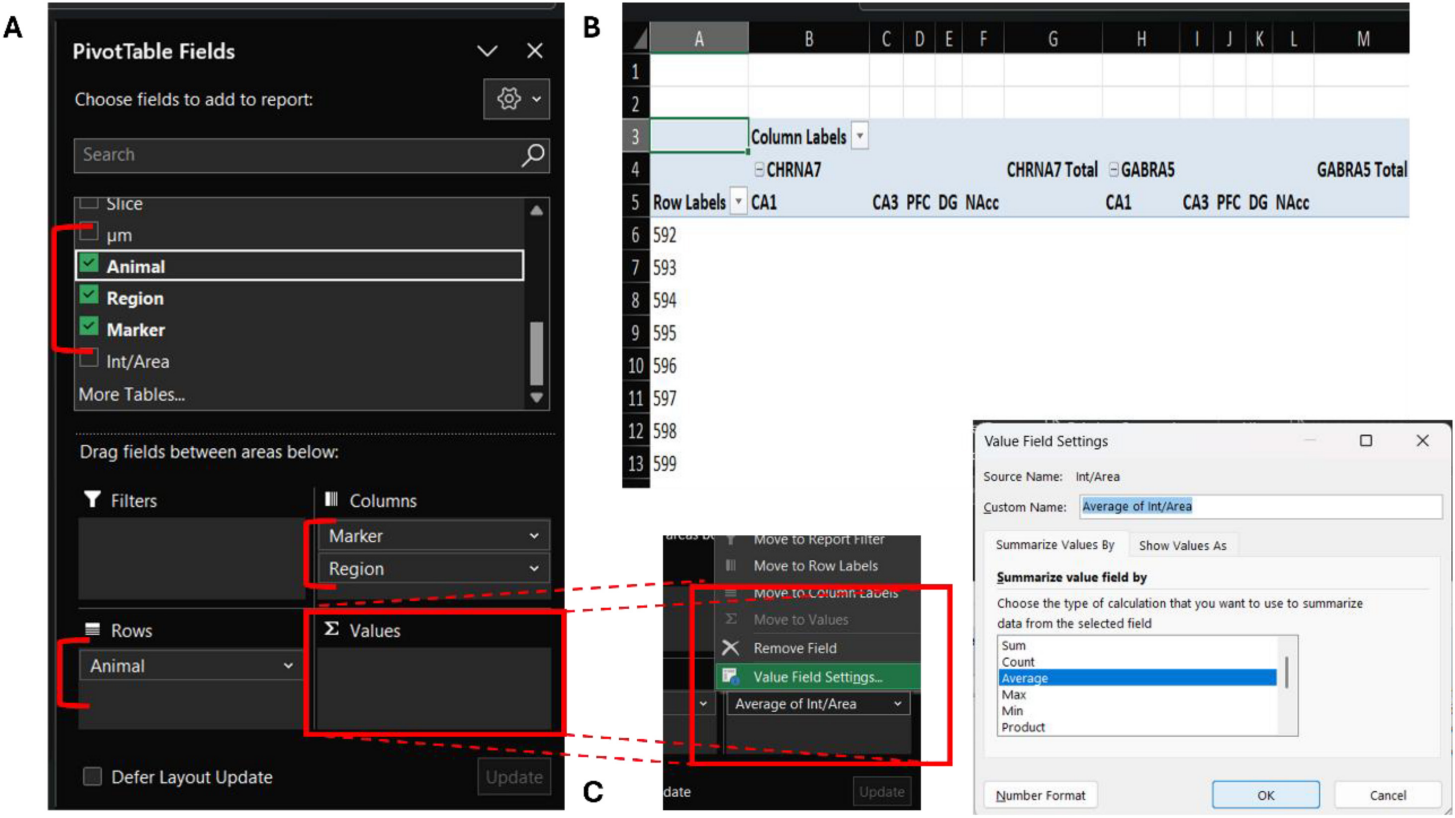
Construction of a PivotTable to organize optical density measurements across animals, regions, and markers. The PivotTable field interface **(A)** allows the selection and placement of dataset variables into the table structure. In this example, Animal (Level 2) is assigned to the Rows field, whereas Region (Level 3) and Marker (Level 4) are assigned to the Columns field **(B)**. The calculated Int/Area variable is placed in the Values field to summarize the normalized intensity for each condition. The resulting PivotTable (top right) displays the hierarchical organization of markers and brain regions across animals. The Value Field Settings dialog (bottom right) is used to define the method of summarizing the Int/Area values, with the Average selected as the most appropriate measure when the number of images per region varies across samples **(C)**.

In the example shown in Figure 4, Level 2 (animal identifier) is assigned to the *Rows* field, while Level 3 (region) and Level 4 (marker) are assigned to the *Columns* field (Figure 4A). Multiple fields can be placed within the same category to organize data hierarchically. In the illustrated example, *Markers* form the first level of columns, which in turn are divided into *Regions* (Figure 4B). The calculated *Intensity/Area* variable is placed in the *Values* field. This field can summarize the data in several ways, including *count*, *sum*, and *average*, which can be defined in the *PivotTable* value settings (Figure 4C).

When the number of images per region is not identical across samples, which is commonly the case, the *average* function provides the most appropriate representation of the intensity/area value.

With the data accordingly displayed in the pivot table, the transfer to a statistical analysis software becomes straightforward.

### 3.2 StarDist data curation

**FIGURE 5:**
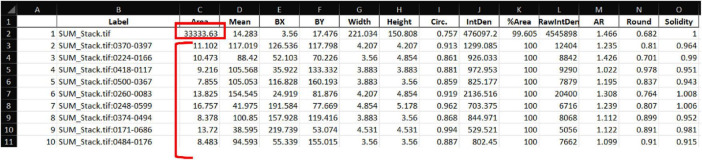
Example of a StarDist output table used for nuclei quantification. The StarDist results file contains two key elements required for downstream cell-density calculations: the total analyzed area and the number of detected nuclei. The total area corresponds to the value in cell C2 (red square), whereas the number of nuclei is obtained by counting all remaining rows in the table, excluding the area entry (red bracket). This structure enables automated extraction of area and cell count values for each sample and region.

The StarDist output is even more straightforward to process and can be fully automated in Python. A script can be used to organize the.*csv* files according to their Level 1 and Level 2 identifier folders (e.g., *182_PFC*, *179_NAcc*). For each folder, the script retrieves the sum of the total area, corresponding to the value in cell *C2* of the *StarDist table* (Figure 5, red square), and the total number of detected nuclei, obtained by counting all remaining entries in the table except the area row (Figure 5, red bracket). This automated consolidation provides, for each animal and region, the total area analyzed and the total number of nuclei detected, enabling subsequent calculation of cell density or normalization factors. Of note, for tables resulting from a double area selection, the area of interest is located in the C3 cell, whereas the C2 area corresponds to the total ROI.

### 3.3 Automated data curation

The Python script provided in the accompanying GitHub repository automates the complete workflow described in section 3.1, from consolidating the individual.*csv* files to generating the final PivotTable-ready dataset. The script concludes by compressing the processed sheets into a single archive for convenient storage. An additional module is included to generate preliminary graphical representations of the data, offering optional visual summaries that allow rapid inspection of trends and data distribution before formal statistical analysis.

## 4 Discussion

Quantitative IF remains an essential approach for examining molecular and cellular changes across brain regions. However, its application often generates large and heterogeneous image datasets that are challenging to process consistently. Traditional fluorescence quantification workflows frequently involve multiple user-driven steps, including ROI delineation, threshold selection, and channel-wise processing. When such parameters are not rigorously standardized, these steps may introduce operator-dependent variability and affect reproducibility, particularly in studies involving multiple targets, anatomical regions, or large sample sizes (Lara et al., 2021; Sanchez-Arias et al., 2021; Shihan et al., 2021). In this context, the present workflow was developed to provide a structured and reproducible implementation of well-established analytical practices, rather than to introduce a novel quantification principle. Importantly, OD measurement using ImageJ/FIJI is a widely accepted and extensively validated approach within the scientific community, with long-standing application across diverse fields of biological imaging (Schindelin et al., 2012; Schroeder et al., 2021). The robustness and flexibility of ImageJ/FIJI, supported by an extensive plugin ecosystem and scripting capabilities, have enabled its adoption for quantitative fluorescence analysis ranging from conventional immunofluorescence to advanced imaging modalities and automated pipelines (Schindelin et al., 2012). By leveraging ImageJ/Fiji for OD quantification in combination with StarDist-based nuclear detection and structured data curation using spreadsheet software and Python, the present workflow consolidates established tools into a coherent pipeline that minimizes user burden while preserving analytical transparency (Schmidt et al., 2018). This integration supports the generation of high-quality, reproducible datasets without altering the underlying quantification principles that are already well-recognized in the literature (Schindelin et al., 2012; Schroeder et al., 2021).

Manual cell counting, including unbiased stereological approaches, is often regarded as a reference method for estimating cell numbers in histological material; however, these techniques are inherently labor-intensive, time-consuming, and dependent on user decisions, which limits scalability for large datasets and can introduce inter- and intra-observer variability, particularly in regions with ambiguous nuclear morphology (Mouton et al., 2017; West, 1999). These practical constraints have driven the increasing adoption of automated and semi-automated image analysis methods. In this context, deep learning-based nuclear segmentation tools such as StarDist have been extensively validated for DAPI-stained and immunofluorescence images, showing strong agreement with manual annotations and enabling robust segmentation across large image sets (Schmidt et al., 2018). Moreover, comparative studies have reported that deep learning–based localization/ segmentation approaches, including StarDist, can achieve performance comparable to classical reference frameworks, including stereology-based estimates, while substantially reducing analysis time and operator-dependent variability (Checcucci et al., 2024). The pipeline presented here adopts this strategy to prioritize standardization, reproducibility, and analytical throughput across large image sets. While this approach may preclude correction of isolated ambiguous objects that could be addressed through manual intervention, it provides a consistent and scalable framework for population-level analyses, in which relative differences in cell density and optical density between experimental groups are the primary endpoints.

A key advantage of this workflow is its balance between automation and user control. Macro-driven steps ensure that critical procedures, such as scale application, ROI selection, and focal-plane determination, are standardized across all images. The structured file-naming strategy preserves contextual metadata and facilitates downstream data curation. The integration of StarDist enables reproducible and objective nuclear detection, reducing subjectivity associated with manual or semi-quantitative counting approaches (Schmidt et al., 2018). In addition, the possibility to transition seamlessly between spreadsheet-based and Python-based data processing provides flexibility for users with different levels of computational expertise, while ensuring that large datasets remain tractable.

Some limitations should nonetheless be acknowledged. The workflow still requires user input during ROI definition and focal-plane selection, which may introduce minor operator-dependent variation, particularly in complex anatomical regions or images with low signal-to-noise ratios. Furthermore, while the pipeline substantially reduces processing time, visual inspection of intermediate outputs remains necessary to ensure data quality, especially in cases involving dual-region analysis. Finally, as with all quantitative imaging approaches, the accuracy of the results depends on consistent acquisition parameters, underscoring the importance of rigorous imaging practices. Despite these considerations, the workflow provides a robust and accessible approach that improves reproducibility and transparency in immunofluorescence quantification. This approach is consistent with well-established and widely accepted practice, in which IF serves as a semi-quantitative tool to compare relative differences in signal or expression density rather than to infer absolute protein abundance (Lara et al., 2021; Majumder and Fisk, 2014). Accordingly, the manuscript does not assume a 1:1 linear relationship between pixel intensity and protein concentration as a result of the current methodology, an assumption that requires complex calibration against molecular standards or orthogonal methods such as mass spectrometry (Sweat et al., 2025; Toki et al., 2017), which was not employed in the present protocol. The pipeline assumes a strict standardization to ensure technical comparability and reproducibility. In the methodological example presented, all samples were processed in parallel using identical fixation, permeabilization, and staining protocols, including the same antibody batches and image acquisition parameters (see Supplementary Table 4). Image pre-processing steps, such as background correction and normalization, should also be applied uniformly, as the pipeline assumes minimized user-dependent interventions to reduce user bias. Potential confounding factors such as autofluorescence and noise should also be addressed within this standardized framework, incorporating background subtraction based on negative controls or tissue-free regions to limit the influence of autofluorescence and noise (Lara et al., 2021; Shihan et al., 2021). In the present methodological example, a z-stack image acquisition was employed. Although this is not a usual method, it is a better way to minimize signal instability due to photobleaching, when used in combination with the selection of stable fluorophores and optimization of exposure times. Under such controlled conditions, photobleaching is generally not a dominant source of variability during comparative imaging sessions. Quantitative outputs are deliberately restricted to relative optical density and cell density metrics, avoiding over-interpretation of intensity values.

Building on these considerations, it is important to position this workflow within the context of existing protocols for quantitative immunofluorescence, many of which rely on similar methodological principles but differ substantially in their degree of automation and user dependency. Most published applications, including those described in recent methodological and biological studies (Régnier et al., 2024; Stansak et al., 2024; Unger et al., 2025), follow a common structure involving ROI definition, threshold-based segmentation, integrated density measurements, and DAPI-based nuclear counting. These approaches have been widely adopted due to their conceptual simplicity and compatibility with standard laboratory imaging practices. Although such workflows can achieve reproducible results when analytical parameters are predefined and consistently applied, many implementations still involve repeated user-defined steps, including ROI delineation, threshold selection, and iterative channel processing, which may introduce operator-dependent variability when strict standardization is not enforced. More systematized versions of these approaches have been assembled into structured protocols, such as the STAR Protocol for fluorescence quantification (Choi et al., 2025), which emphasize best practices for acquisition and normalization. Nevertheless, core analytical steps, including ROI selection, threshold adjustment, and iterative measurement, remain manual, constraining throughput and increasing susceptibility to user-dependent bias. Hybrid pipelines such as PUPAID further extend these workflows by integrating ImageJ with R to enhance reproducibility (Régnier et al., 2024), but at the cost of increased technical complexity and steeper learning curves.

A direct comparison between conventional manual quantification and the macro-based pipeline highlights the practical impact of automation (Supplementary information 3). In ImageJ workflows, manual analysis of fluorescence channels from the same image must be processed independently, requiring repeated image import, focal-plane selection, z-projection, ROI handling, and measurement steps. Results are not saved automatically and typically require manual file naming, increasing processing time and the risk of labeling inconsistencies, while embedded metadata is limited and not customizable. Consistent with this complexity, in our lab, two independent users required at least 37 min each, to quantify four standard images, with no dual area selection or total area correction (≈9.3 min/image), and 1 h 40 min to quantify seven complex images, with total area corrections and dual-area selection (≈14.3 min/image). In contrast, using the macro-based workflow, 13 images with standard parameter requirements were processed in 9 min (≈0.7 min/image), while 7 more complex images, including area adjustments and dual-region analyses, were processed in 12 min (≈1.7 min/image). These results correspond to an approximately 5–20-fold reduction in processing time per image compared with manual quantification. These differences are summarized in Table 1, which compares the operational characteristics of manual and macro-based workflows. The observed improvement reflects the consolidation of multi-channel analysis into a single processing sequence, automated data export with standardized, metadata-rich labeling, and reduced user intervention. Importantly, these gains are achieved without altering the underlying quantification principles.

**TABLE 1:** Comparison between manual and acro-based immunofluorescence quantification workflows.

Feature	Manual ImageJ workflow	Macro-based workflow
Channel processing	Each channel is processed independently	All selected channels are processed in a single sequence
Number of user interactions	High (multiple menu operations per image and channel)	Low (guided prompts only)
ROI handling	Manual creation, saving, loading, and inversion	Single ROI definition with optional multi-area support
DAPI quantification	Grid-based subsampling and extrapolation	Full-image automated nuclear detection (StarDist)
Data saving	Manual export and file renaming	Automatic export with standardized labeling
Metadata structure	Limited and non-customized	Metadata-rich, derived from the directory hierarchy
Processing time (min/image)	9.3–14.3	∼0.7–1.7
Inter-operator variability	High	Minimal
Scalability	Limited for large datasets	Suitable for high-throughput analysis

In this context, the similarity between the present workflow and established methods should be viewed as a strength rather than a limitation. Because the analytical framework, threshold-based segmentation, integrated density quantification, and DAPI-based nuclear counting, match approaches that have been extensively validated in the literature (Sanchez-Arias et al., 2021; Stansak et al., 2024; Unger et al., 2025), results generated with this pipeline remain directly comparable to existing datasets. The contribution of this work, therefore, lies not in redefining immunofluorescence quantification but in delivering a streamlined, reproducible, and scalable implementation of an accepted methodology, better suited to contemporary experimental designs involving large and complex imaging datasets.

In summary, this work presents a streamlined and reproducible workflow for quantitative immunofluorescence analysis that preserves the analytical foundations of widely accepted manual protocols while substantially improving efficiency, consistency, and scalability. By consolidating image processing, nuclear quantification, and OD measurement into a guided macro-based pipeline, the method reduces user-dependent variability and analytical burden without requiring specialized computational expertise. This balance between methodological familiarity and operational improvement makes the workflow particularly suitable for studies involving large imaging datasets, multiple markers, and complex experimental designs, supporting more transparent and reproducible quantitative fluorescence analyses.

## Data Availability

The datasets presented in this study can be found in online repositories. The names of the repository/repositories and accession number(s) can be found in the article/Supplementary material.

## Footnotes

1
https://imagej.net

2
https://github.com/up246512-ui/Elmo-pipeline-public/blob/main/macro/ElmFluoLzCount.ijm

3
https://github.com/up246512-ui/ElmFluoLzCount-pipeline-public
