# Supplementary material for: A semi-automated pipeline integrating ImageJ/Fiji and StarDist for the reproducible quantification of cellular and optical density metrics in immunofluorescence images of brain tissue
Source: Front Neuroanat. 2026 Mar 27;20:1778296. doi: 10.3389/fnana.2026.1778296 (PMC13066318; doi:10.3389/fnana.2026.1778296)
Supplement: Supplementary file 1 [file Data_sheet_1.pdf]

- Supplementary Information -

Sandra Isabel Marques[1,2], Helena Carmo[1,2], Félix Carvalho[1,2], João Pedro Silva[1,2,#], Susana Isabel Sá[3,#]

[1]UCIBIO – Applied Molecular Biosciences Unit, Laboratory of Toxicology, Faculty of Pharmacy, University of Porto, 4050-313 Porto, Portugal;

[2]i4HB – Institute for Health and Bioeconomy, Faculty of Pharmacy, University of Porto, 4050-313 Porto, Portugal;

[3]RISE-HEALTH, Unit of Anatomy, Department of Biomedicine; Faculty of Medicine, University of Porto, 4200-319 Porto, Portugal;

[#] Co-senior and corresponding authors:

João Pedro Silva, Associate Laboratory i4HB - Institute for Health and Bioeconomy, UCIBIO, Laboratory of Toxicology, Department of Biological Sciences, Faculty of Pharmacy, University of Porto, 4050-313, Porto, Portugal, Tel: +351 220428796;

Susana I. Sá, RISE-HEALTH, Unit of Anatomy, Department of Biomedicine; Faculty of Medicine, University of Porto, 4200-319 Porto, Portugal. Tel: +351 220426552.

E-mails: jpmsilva@ff.up.pt (JPS), sasusana@med.up.pt (SIS)

Table SI-1 Comparison of representative fluorescence quantification workflows and the present pipeline

Feature	AutoCount (Sharara et al., 2025)	ACCT (Kataras et al., 2023)	Lusca (Šimunić et al., 2024)	Present Workflow
**Primary objective**	Automated fluorescent cell counting	ML-based cell counting in Fiji	Morphological analysis of cellular/subcellular structures	Reproducible extraction of nuclear density and optical density (OD) metrics
**Core segmentation strategy**	ImageJ macro-based automated counting	Trainable Weka Segmentation (user-trained ML classifier)	ML-based segmentation with extensive morphometric analysis	Pretrained StarDist deep-learning model (DAPI nuclear segmentation)
**Model training required**	No	Yes (classifier training per dataset)	Yes (classifier tuning required)	No (fixed pretrained model applied uniformly)
**Scope of outputs**	Cell counts	Cell counts + object features	Broad morphometrics (area, volume, branching, etc.)	Nuclear counts/density + ROI-level integrated optical density
**Multi-channel handling**	Typically single-channel per run	Channel-wise processing	Channel-specific segmentation and measurement	Simultaneous multi-channel OD quantification
**Z-stack handling**	User-dependent preprocessing	User-dependent preprocessing	Supports 2D/3D analysis; preprocessing assumed	Explicit focal-range selection + standardized stack handling
**Metadata/export strategy**	Standard ImageJ outputs	Standard Fiji/ImageJ outputs	Fiji outputs; not centered on metadata structuring	Directory-derived identifiers + structured export +

				pivot/Python consolidation
**Validation approach**	Compared to manual counting	Compared to manual counts	Compared to manual morphometrics	Manual vs StarDist validation with correlation and relative error metrics
**Design emphasis**	Speed of counting	Flexible ML segmentation	Comprehensive morphological profiling	Workflow standardization, reproducibility, and scalability

Table SI-1 represents a comparison of representative fluorescence quantification tools with the present ImageJ/Fiji + StarDist workflow. Recently published Fiji/ImageJ-based tools, including macro-based automated counting (AutoCount), machine-learning–based segmentation using Trainable Weka Segmentation (ACCT), and broader morphometric analysis frameworks (Lusca), are contrasted with the present workflow in terms of segmentation strategy, model training requirements, scope of outputs, metadata handling, validation framework, and design emphasis. The table highlights that the present study does not introduce a novel segmentation algorithm, but rather integrates established tools into a standardized, metadata-preserving pipeline optimized for reproducible extraction of nuclear density and optical density metrics from large immunofluorescence datasets.

Validation of automated nuclear detection against manual counting

To evaluate the performance of the automated StarDist-based nuclear detection, cellular density estimates were compared against manual grid-based counts performed on a representative subset of DAPI-stained images across multiple

brain regions. Manual counting was conducted by a trained operator under predefined criteria regarding focal-plane inclusion, exclusion of truncated nuclei at image borders, and separation of closely apposed nuclei when distinct morphological boundaries were visible, as detailed in Supplementary Information (Manual Quantification). Only nuclei clearly in focus within the selected imaging plane were considered. Manual counts were converted to cellular density (cells/mm^2) by normalizing the total number of nuclei to the analyzed area, thereby matching the output format of the automated pipeline. Automated quantification was conducted independently using fixed StarDist parameters applied uniformly across the dataset, and automated outputs were not modified based on manual observations.

A schematic comparison between sampling-based manual counting and automated full-ROI nuclear detection is illustrated in Figure SI-1, where manual estimation of areal density (Na) is derived from systematic grid sampling within the region of interest (ROI), whereas the automated approach performs full-ROI nuclear quantification. Representative examples highlighting practical limitations of manual sampling in high-density or anatomically heterogeneous regions are shown in Figures SI-2 and SI-3. These examples illustrate how sampling-based approaches may require substantial effort to capture local spatial heterogeneity and remain sensitive to sampling variability, particularly in regions such as CA1, CA3, and the dentate gyrus.

Quantitative agreement between manual and automated measurements is presented in Figure SI-4, which displays image-by-image scatter plots comparing cellular density estimates across analyzed brain regions. Agreement was assessed using Pearson’s correlation coefficient (r) to evaluate linear association,

Spearman's rank correlation coefficient (ρ) to assess monotonic agreement independent of strict linearity assumptions, and mean absolute percentage error (MAPE) to quantify differences in absolute magnitude between methods. Detailed region-specific statistics, including Pearson's r, Spearman's ρ, and MAPE values, are reported in Table SI-2.

Image-by-image comparisons demonstrated a positive association between manual and automated density estimates across all regions. Pearson correlations indicated strong linear agreement in CA3 and prefrontal cortex (PFC) and moderate agreement in CA1 and dentate gyrus, with Spearman correlations showing comparable trends. Although correlation coefficients confirmed preservation of spatial trends between methods, MAPE values revealed differences in absolute magnitude between sampling-derived manual estimates and automated full-ROI measurements.

These discrepancies are consistent with fundamental methodological differences between the approaches. Manual grid-based counting provides a sampling-derived estimate of cellular density and is therefore inherently sensitive to local spatial heterogeneity. In contrast, the automated StarDist-based method quantifies all detectable nuclei within the defined ROI, effectively performing a census of the region. By reducing sampling-related variability, the automated pipeline yields more consistent density measurements across images, particularly in high-density and structurally heterogeneous regions. These analyses were performed to contextualize the performance of the automated workflow relative to manual counting, rather than to establish manual quantification as an absolute reference standard.

Figure SI-1. Comparative analysis of manual grid-based and automated StarDist-based methods for DAPI nuclear density estimation. Comparison of

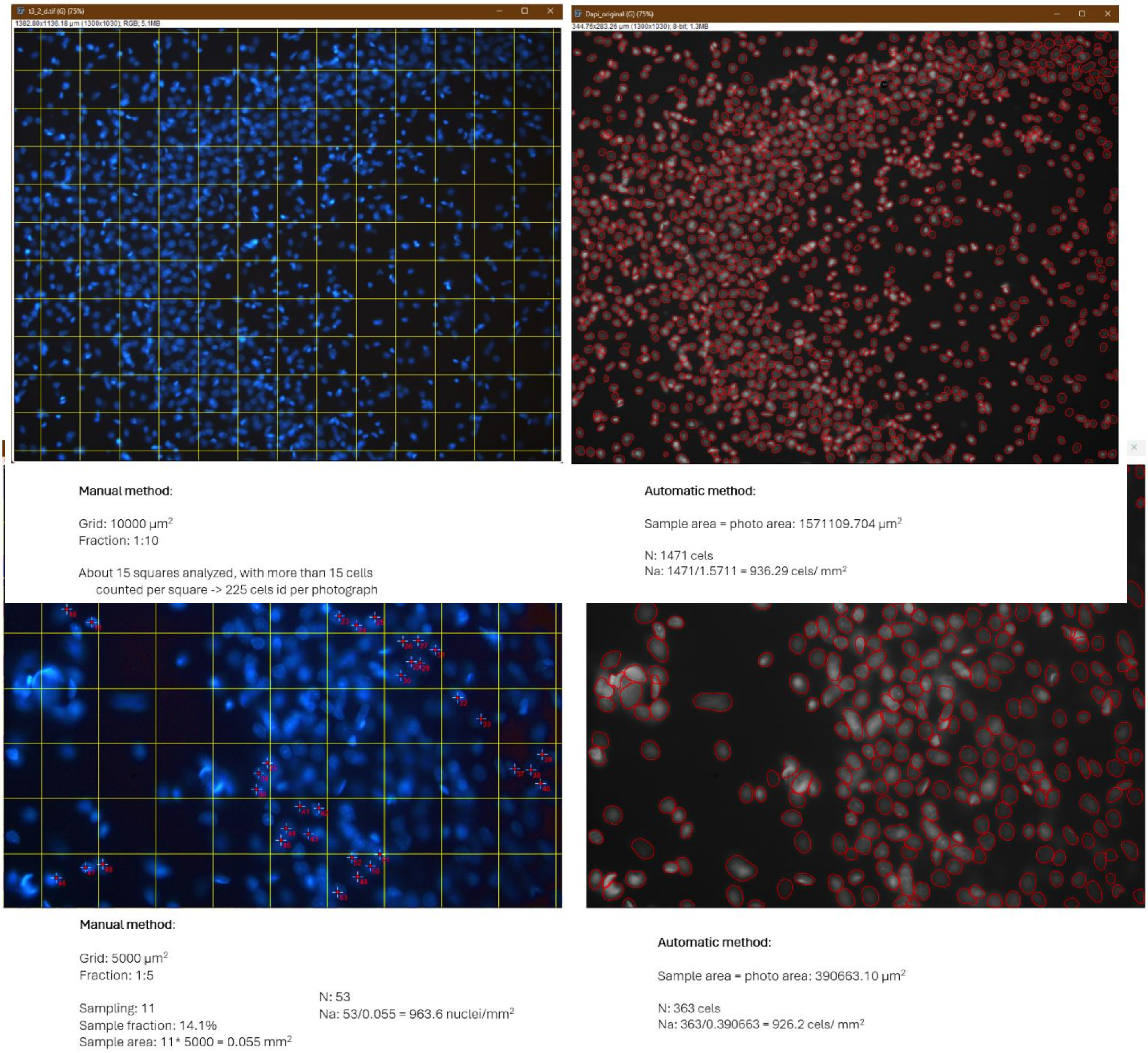

manual (grid-based) and automated (StarDist) methods for determination of areal density of DAPI-stained nuclei. Left panel, manual method with superimposed grid and manual count of cells in a 1:5 sampling scheme. Calculations of Na (cell density/area) in each method are presented. Photomicrographs taken from one optical plane with a 20x objective.

Figure SI-2. Illustration of manual sampling limitations in high-density fields and corresponding automated full-field nuclear detection using StarDist. When cell density is high, the manual method is not feasible, hence an automated method is preferred. Photomicrographs taken from one optical plane with a 10x objective.

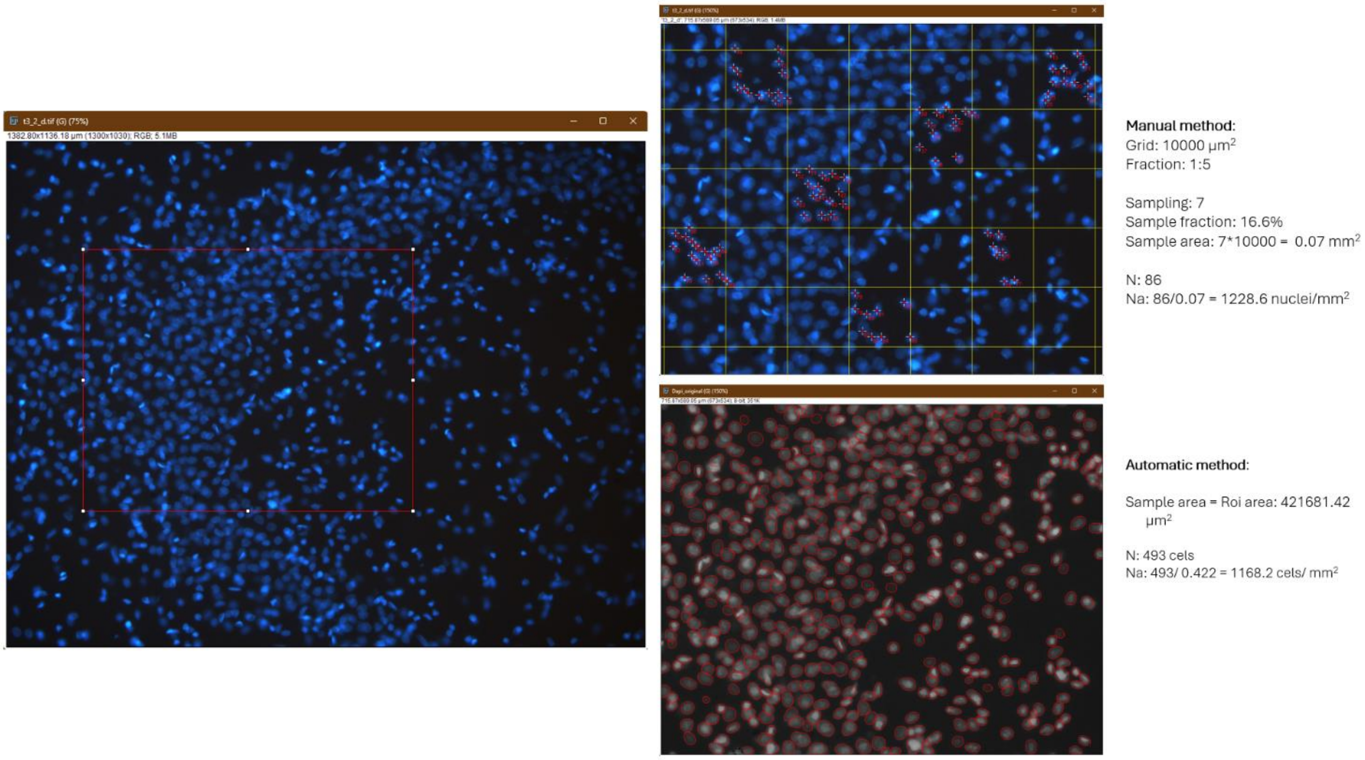

Figure SI-3. **ROI-restricted manual sampling in high-density fields and comparison with automated full-field nuclear detection.** When cell density is high, the manual method requires the determination of a ROI for better usage of the method. This makes the method more feasible but increases the error. Photomicrographs taken from one optical plane with a 10x objective.

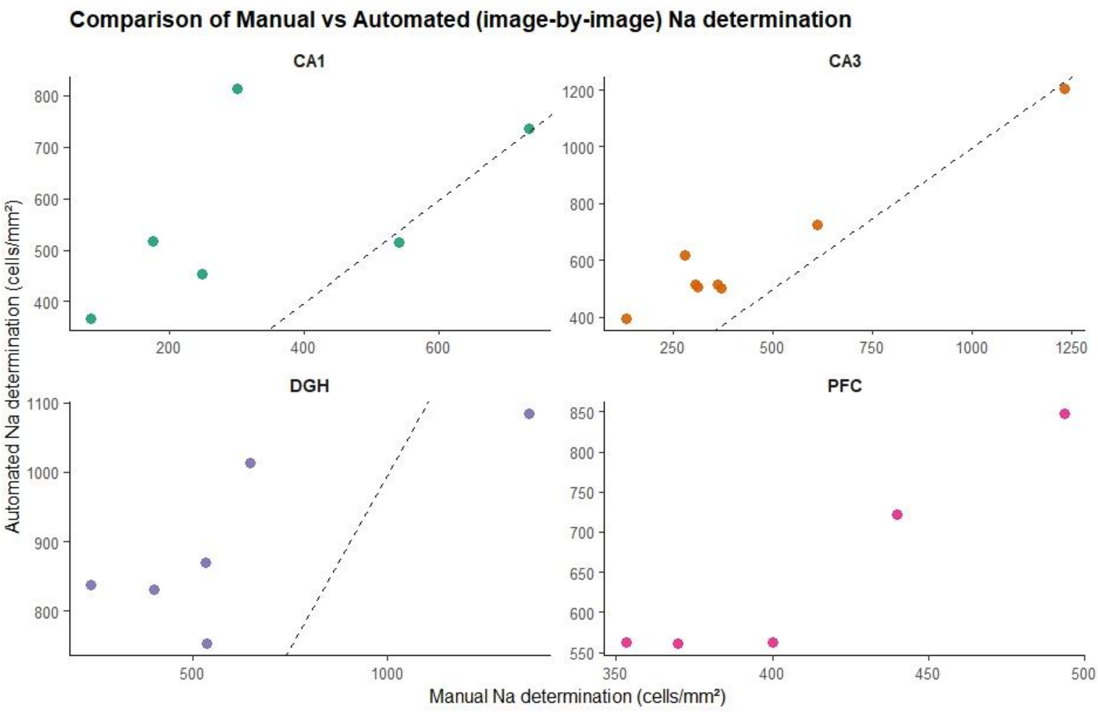

Figure SI-4. Manual versus automated cellular density estimates across brain regions. Scatter plots show an image-by-image comparison between manually derived cellular density (cells/mm²) and StarDist-based automated cellular density for each brain region. Each point represents a single image. The dashed line indicates the identity line (y = x). Differences between methods reflect the comparison between sampling-based manual estimates and automated measurements derived from analysis of the full region of interest, particularly in regions with heterogeneous cellular distributions.

Table SI-2. **Comparison between manual and automated cellular density estimates.**

Number of images (n), Pearson's correlation coefficient (r) that measures the strength and direction of the linear relationship between two continuous variables, with values range from −1 to +1, with values close to ±1 indicating a strong linear association and values near 0 indicating weak or no linear relationship. In general, |r| ≥ 0.7 is

considered strong, 0.4–0.7 moderate, and <0.4 weak. In this context, Pearson's r reflects how closely the automated measurements vary proportionally with manual estimates. **Spearman's rank correlation coefficient (ρ)** assesses the strength of a monotonic relationship between two variables based on ranked values rather than raw measurements. Like Pearson's r, it ranges from −1 to +1, with similar interpretative thresholds (|ρ| ≥ 0.7 strong, 0.4–0.7 moderate, <0.4 weak). Because it is based on ranks, Spearman's ρ is less sensitive to outliers and does not assume a strictly linear relationship. And **Mean Absolute Percentage Error (MAPE)** quantifies the average magnitude of disagreement between two measurement methods, expressed as a percentage of the manual reference values. Unlike correlation coefficients, which describe association in trend, MAPE reflects differences in absolute magnitude between measurements; are reported for the image-by-image comparison, in each brain region, between manual and automated cellular density measurements.

**Area**	**N**	**Pearson (r)**	**Spearman (p)**	**MAPE**
**CA1**	6	0.55	0.60	131.60
**CA3**	8	0.98	0.60	68.56
**DGH**	6	0.80	0.60	89.59
**PFC**	5	0.96	0.82	57.50

Image-by-image comparisons showed a positive association between manual and automated, StarDist-based cellular density estimates across all regions. Pearson correlations were strong in CA3 and PFC and moderate in CA1 and DGH, indicating that both methods capture similar spatial trends in cellular density. Despite these correlations, mean absolute percentage error (MAPE) values were high in all regions, revealing substantial differences in the absolute

magnitude of the density estimates. These discrepancies were more pronounced in areas with marked anatomical heterogeneity and limited sample size. This pattern reflects fundamental methodological differences between the two approaches. Manual grid-based counting provides a sampling-based estimate of cellular density, making it more sensitive to local density fluctuations. In heterogeneous regions such as CA1, CA3, and the dentate gyrus, this sensitivity likely contributes to elevated MAPE values even when correlations remain preserved. In contrast, the StarDist-based method analyzes the entire ROI, effectively providing an exhaustive density estimate. By reducing sampling-related variability, the automated approach yields more consistent density measurements across images. This characteristic is particularly advantageous for large datasets and for studies that rely on robust comparative analyses across regions or conditions.

Mitigation tactics to standardize the analysis process

Table SI-3 - Common sources of variability in quantitative immunofluorescence and corresponding mitigation strategies implemented in the present workflow.

Known immunofluorescence limitation	Mitigation strategy adopted
Non-stoichiometric antibody–antigen binding	Quantification interpreted as relative signal intensity rather than absolute abundance; comparisons restricted to samples processed and acquired under identical conditions
Variable tissue permeability	Use of free-floating sections, standardized thickness, detergent-containing incubation buffers, and consistent incubation times across samples

Known immunofluorescence limitation	Mitigation strategy adopted
Epitope accessibility	Optimization of fixation protocol; consistent antigen retrieval strategy (or explicit avoidance); uniform processing across all groups
Antibody affinity and specificity	Use of validated antibodies; identical antibody batches per experiment; inclusion of negative controls; emphasis on relative comparisons; processing an equal number of animals from each group at the same time in order to ensure that they share the same environmental conditions of temperature and humidity
Autofluorescence	Consistent background handling; ROI-based OD quantification; reliance on integrated density rather than threshold-based segmentation; use of autofluorescence quenching kits, such as TrueVIEW® Autofluorescence Quenching kit with DAPI (#SP-8500) acquired from Vector Laboratory Inc. (Newark, USA) or Sudan Black Blue
Photobleaching	Minimized exposure time; identical acquisition settings across samples (namely with z-stack capture); avoidance of repeated scanning
Channel bleed-through / spectral overlap	Predefined filter sets; fixed channel assignments; simultaneous multi-channel processing without post hoc reassignment
Inconsistent image acquisition parameters	Strictly fixed acquisition parameters per marker; exclusion of cross-session normalization
Z-stack focal-plane selection variability	Explicit user-guided focal-range selection followed by standardized stack handling

Table SI-3 summarizes well-recognized biological, biochemical, and technical limitations inherent to immunofluorescence analysis, including non-stoichiometric antibody-antigen interactions, tissue permeability, epitope accessibility, autofluorescence, and image acquisition-related factors, and outlines the

procedural and analytical measures adopted to minimize their impact on OD and cellular density quantification. These strategies are based on established best practices and are intended to improve reproducibility and consistency without altering the underlying principles of immunofluorescence quantification.

Comparison between manual and macro-based immunofluorescence quantification workflows

Table SI-4 - Step-by-step comparison between manual and macro-based immunofluorescence quantification workflows.

Manual quantification workflow	Macro-based quantification workflow
**Create output folder hierarchy (Target → Animal → Area)**	Launch macro plugin
**Import image sequence for channel c0**	Select the input directory, and the output establishment
**Manually browse and filter files by channel**	Define analysis parameters (channels, regions, scale)
**Generate a montage to inspect the z-stack**	Display tiled stack for focus inspection
**Adjust brightness/contrast for visualization**	
**Select in-focus slices manually**	Select first and last in-focus slices
**Run Slice Keeper**	-

Manual quantification workflow	Macro-based quantification workflow
**Perform z-projection (sum slices)**	-
**Manually create ROI (polygon/rectangle)**	Define ROI (optional multi-area definition)
**Save ROI to disk**	-
**Run StarDist on DAPI image**	Confirm ROI for StarDist execution
**Measure and export DAPI results**	-
**Manually name and save results file**	-
**Re-import image sequence for channel c1**	-
**Repeat Slice Keeper and z-projection**	-
**Load previously saved ROI**	-
**Measure optical density**	Confirm ROI for OD quantification
**Re-import image sequence for channel c2**	-
**Repeat Slice Keeper and z-projection**	-
**Reload ROI and measure optical density**	-
**Manually save and rename the OD results file**	-
**For dual-region images, manually delineate subregions**	Optional definition of multiple ROIs
**Save, invert, reload ROIs repeatedly**	-

Manual quantification workflow	Macro-based quantification workflow
**Repeat StarDist and OD measurements per subregion**	-
**Close all windows**	-
**Repeat the full workflow for the next image**	Automatically iterate steps 4–8 for all images
**Manually count DAPI nuclei using grid sampling (optional)**	Automatic full-image nuclear counting (StarDist)
**Manually rename and save all remaining files**	Automatic saving of all outputs with standardized metadata

This table provides a side-by-side comparison of the sequential steps required to perform quantitative immunofluorescence analysis using a conventional manual ImageJ workflow and the macro-based pipeline presented in this study. The manual workflow reflects a standard ImageJ-based approach in which each fluorescence channel is processed independently, and multiple user-driven operations are required for focal-plane selection, region-of-interest (ROI) definition, segmentation, measurement, and data export. In contrast, the macro-based workflow consolidates these operations into a reduced number of guided steps, enabling simultaneous multi-channel processing, automated execution of nuclear detection and optical density quantification, and systematic saving of output files with standardized, metadata-rich labeling.
